# Type I-E CRISPR-Cas System as a Defense System in Saccharomyces cerevisiae

**DOI:** 10.1128/msphere.00038-22

**Published:** 2022-04-27

**Authors:** Gargi Bindal, Lina Amlinger, Magnus Lundgren, Devashish Rath

**Affiliations:** a Applied Genomics Section, Bhabha Atomic Research Centre, Mumbai, India; b Homi Bhabha National Institute, Mumbai, India; c Department of Cell and Molecular Biology, Uppsala University, Uppsala, Sweden; University College Dublin

**Keywords:** CRISPR-Cas, Cascade, interference, *Saccharomyces cerevisiae*, type I-E

## Abstract

Defense against viruses and other mobile genetic elements (MGEs) is important in many organisms. The CRISPR-Cas systems found in bacteria and archaea constitute adaptive immune systems that can acquire the ability to target previously unrecognized MGEs. No CRISPR-Cas system is found to occur naturally in eukaryotic cells, but here, we demonstrate interference by a type I-E CRISPR-Cas system from Escherichia coli introduced in Saccharomyces cerevisiae. The designed CRISPR arrays are expressed and processed properly in S. cerevisiae. Targeted plasmids display reduced transformation efficiency, indicative of DNA cleavage.

**IMPORTANCE** Genetic inactivation of viruses and other MGEs is an important tool with application in both research and therapy. Gene editing using, e.g., Cas9-based systems, can be used to inactivate MGEs in eukaryotes by introducing specific mutations. However, type I-E systems processively degrade the target which allows for inactivation without detailed knowledge of gene function. A reconstituted CRISPR-Cas system in S. cerevisiae can also function as a basic research platform for testing the role of various factors in the interference process.

## INTRODUCTION

Viruses and other mobile genetic elements (MGEs) are potential threats to most studied cellular organisms, by acting as predators or by reducing fitness. In response, organisms have evolved multiple defense strategies, which are grouped largely into innate and adaptive systems. Innate systems are characterized by being activated by certain preset features of infection. An adaptive system, on the other hand, can learn to detect previously unrecognized pathogens. For a long time, the vertebrate adaptive immune system was the only known example of an adaptive system, but the clustered regularly interspaced short palindromic repeats (CRISPR)-Cas systems of archaea and bacteria have been demonstrated to be bona fide adaptive immune systems (1). All studied CRISPR-Cas systems are based on short DNA or RNA sequences (protospacers) from, e.g., virus genomes being stored as DNA spacers in the CRISPR locus. A long precursor CRISPR transcript (pre-crRNA) is processed into CRISPR RNA (crRNA) and used by Cas protein effectors to locate and destroy matching targets. The target can be DNA or RNA depending on the type of CRISPR-Cas system. CRISPR-Cas systems are very diverse and are currently grouped into two classes. Class 1 includes type I, III, and IV systems and class 2 includes type II, V, and VI systems. Each type of system includes several subtypes (2, 3).

Programmable nucleases, such as zinc-finger nucleases (ZFNs), transcription activator-like effector nucleases (TALENs), and Cas9, can function as anti-MGE systems in eukaryotic cells by inducing crippling mutations. Particularly, Cas9, which has revolutionized gene editing in eukaryotes, has been demonstrated to effectively target several human viruses (4). In basic Cas9 technology, DNA cleavage is directed by a single guide RNA (sgRNA). The break can be repaired by homology-directed repair (HDR) or error-prone nonhomologous end joining (NHEJ) (2). In the case of viruses, if a gene is repaired by the error-prone NHEJ, it may be inactivated, resulting in an inability of the virus to proliferate, as demonstrated for, e.g., HIV-1 (5). However, this approach requires a thorough understanding of virus biology, as randomly targeting a virus gene does not guarantee reduced proliferation.

In this study, we take advantage of the properties of the type I-E CRISPR-Cas system to test its ability to function as an anti-MGE system in eukaryotes that do not require understanding of target gene function. Type I-E is a class 1 CRISPR Cas system that includes Cascade, Cas3, and the CRISPR locus. Cascade is a multisubunit protein-RNA complex, and Cascade from Escherichia coli consists of five different proteins, namely, Cse1, Cse2, Cas7, Cas5, and Cas6e (in 1, 1, 6, 1, and 2 copies, respectively) and 1 61-nucleotide (nt) crRNA molecule (6) The target of Cascade is recognized by the variable spacer sequence in the crRNA (7) and the presence of a protospacer adjacent motif (PAM) in the target (8). Once a target is identified, the Cas3 effector is recruited to degrade the target (9). Unlike the DNA-targeting class 2 effectors, such as Cas9 and Cas12, which perform blunt (10) and staggered double-stranded cut (11), respectively, Cas3 is an endonuclease which destroys the target in a processive manner (9). Type I CRISPR-Cas systems have been used in human cells to introduce large genomic deletions at specific locations (12–14), and by using Cascade-FokI-fusions, gene editing has been achieved (13). The processive DNA degradation by Cas3 could also make the system highly suitable in applied use for removing virus genomes, as repair that restores virus function is less likely to occur. Since the effect should be the same irrespective of target gene function, Cascade-Cas3 could also be used against poorly characterized viruses. Establishment of a type I-E system in Saccharomyces cerevisiae is also beneficial for basic research, as it allows one to test the role of different factors on CRISPR-Cas immunity in a convenient model system, independent of their original genetic background.

As a proof of concept, we adapted the type I-E system from E. coli for use as a programmable anti-MGE system in Saccharomyces cerevisiae, which was chosen for its role as a eukaryotic model system. We use plasmids as targets, which allow a comparison with similar experiments in bacterial systems (10, 15–17). As a eukaryotic organism, S. cerevisiae does not contain any natural CRISPR-Cas system, and it also lacks RNA interference (RNAi) systems (18). Several viruses of S. cerevisiae are known but none studied have an extracellular stage and mostly spread thorough cell-cell contacts. Certain genetic features may protect S. cerevisiae cells from a specific virus, but there are no known general immune systems (19).

## RESULTS

### Design and reconstitution of the type I-E CRISPR-Cas system in S. cerevisiae.

**FIG 1:**
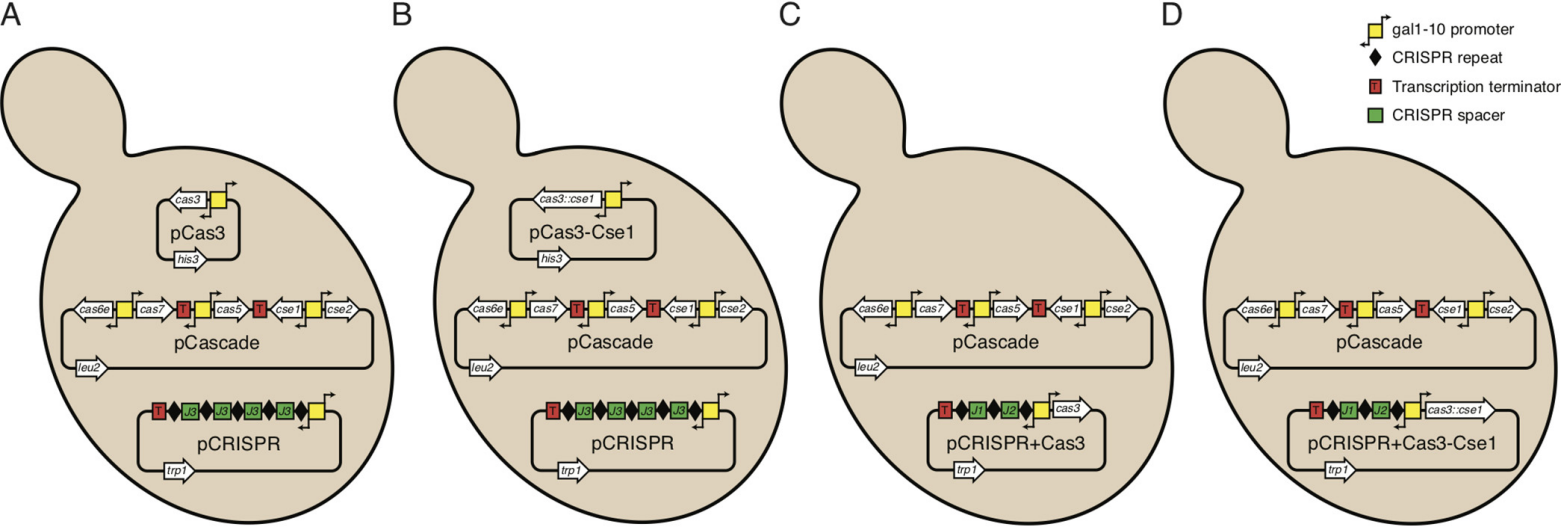
(A to D) The different variants of the type I-E CRISPR-Cas system used for expression in S. cerevisiae.

Our basic system for expressing Cascade, Cas3, and crRNA in S. cerevisiae was different plasmids from the pRSGal series, where each produced one of the components (Fig. 1A), but we also designed several alternative systems (Fig. 1B to D). pCascade, expressing Cascade proteins, had three cassettes with different *cas* genes under the control of bidirectional Gal promoters. The cassettes were separated by a S. cerevisiae CYC1 terminator to prevent formation of antisense transcripts.

For the production of targeting crRNA, pCRISPR was constructed by inserting a CRISPR array containing four copies of the J3 spacer (4×J3) (20) under the control of a Gal promoter, with a CYC1 terminator preventing read-through.

For the expression of the Cas3 nuclease, we designed pCas3 by cloning E. coli *cas3* under a Gal promoter. An alternative version of the plasmid, pCas3-Cse1, carried a naturally occurring *cas3-cse1* fusion (9) (Fig. 1B). Coexpression of this plasmid with pCascade would result in incorporation of the Cas3-Cse1 fusion in a subset of the Cascade complexes. To test the effect of different crRNAs, we combined a CRISPR with two alternative spacers, namely, J1 and J2, with Cas3 or Cas3-Cse1 fusion to generate pCRISPR+Cas3 and pCRISPR+Cas3-Cse1, respectively.

Two different target plasmids were designed. pTargetHigh carried a phage Lambda J gene fragment containing the PAMs and protospacers for J1, J2, and J3 (see Table S1 in the supplemental material) on a high copy vector. pTargetLow carried the same J fragment on a low copy backbone. Original vectors lacking the J fragment were used as nontargeted controls.

10.1128/msphere.00038-22.5 TABLE S1 Sequences of constructs, CRISPRs, and targets. Download Table S1, DOCX file, 0.02 MB. Copyright © 2022 Bindal et al. 2022 Bindal et al. https://creativecommons.org/licenses/by/4.0/ This content is distributed under the terms of the Creative Commons Attribution 4.0 International license.

### Processing of crRNA in S. cerevisiae.

**FIG 2:**
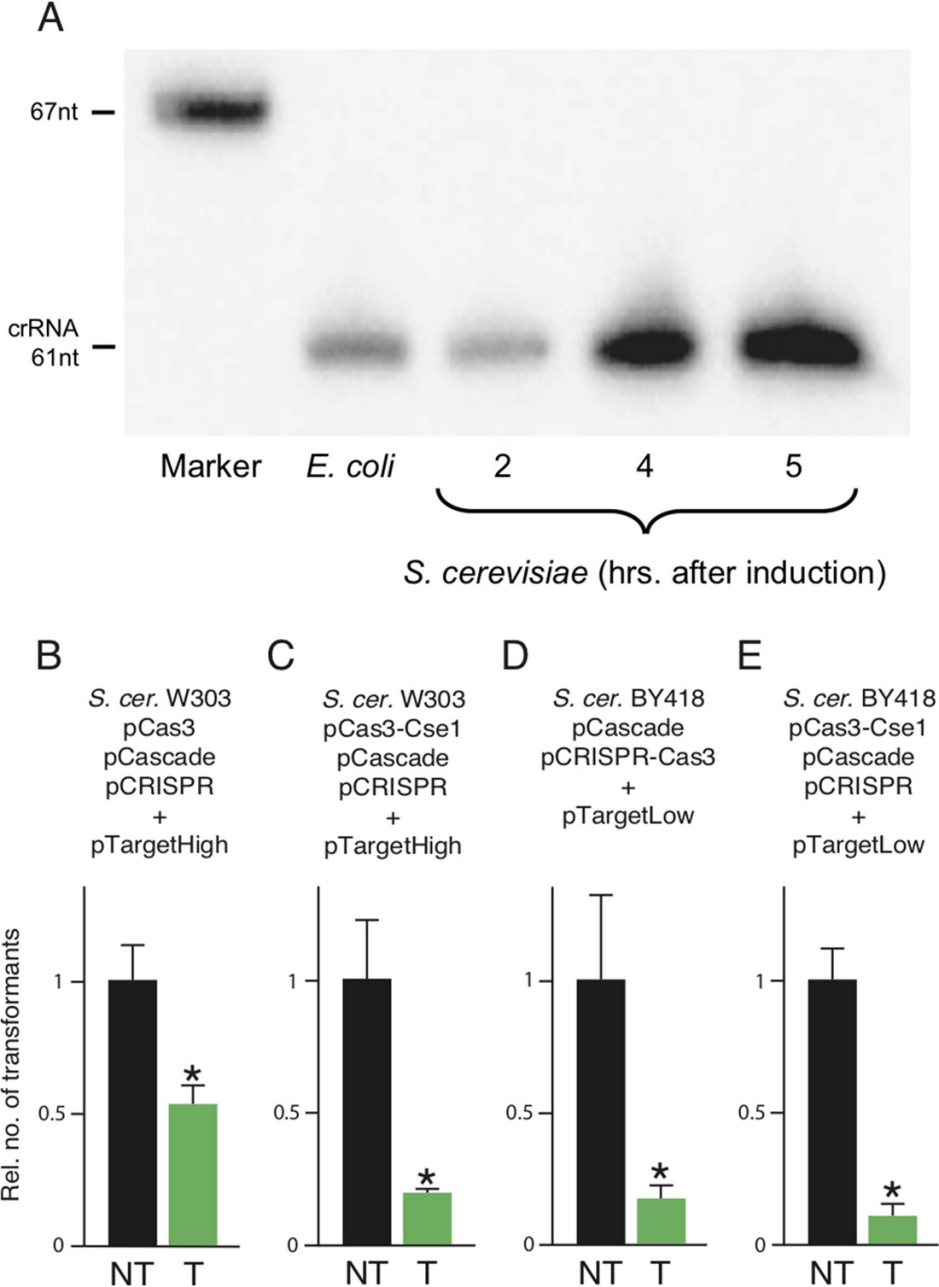
(A) Analysis of crRNA by Northern blotting. Samples of S. cerevisiae W303 carrying pCascade, pCRISPR, and pCas3 were taken at indicated time points after the induction of *cas* genes and CRISPR array expression. RNA from an E. coli strain active for interference was used as the positive control. (B to E) Analysis of CRISPR-Cas interference with plasmids in different S. cerevisiae strains with indicated CRISPR-Cas expression system. Transformation was performed with either with a plasmid containing a target sequence (T) or the nontargeted parent plasmid (NT). The S. cerevisiae strain, target plasmid, and system for CRISPR-Cas expression used are indicated above the graphs. Data in B to E are a summary of three independent biological replicates normalized so that the relative level of transformation by the nontarget plasmid is equal in the different panels. Error bars indicate one standard deviation. A statistically significant reduction of transformation by T compared to NT as determined by Mann-Whitney U test is indicated by an asterisk (*P* ≤ 0.05).

To test the function of crRNA processing in yeast, we analyzed the formation of crRNA in S. cerevisiae W303 after expression of pCascade, pCas3, and pCRISPR was induced by addition of galactose. The analysis was performed by Northern blotting with a radioactively labeled probe complementary to the J3 spacer. The amount of crRNA produced was assayed 2, 4, and 5 h after induction, and crRNA was detected at all time points. The assay demonstrates formation of a 61-nt RNA species, equal in size to crRNA produced in Escherichia coli (Fig. 2A).

### Interference by CRISPR-Cas in S. cerevisiae.

**FIG 3:**
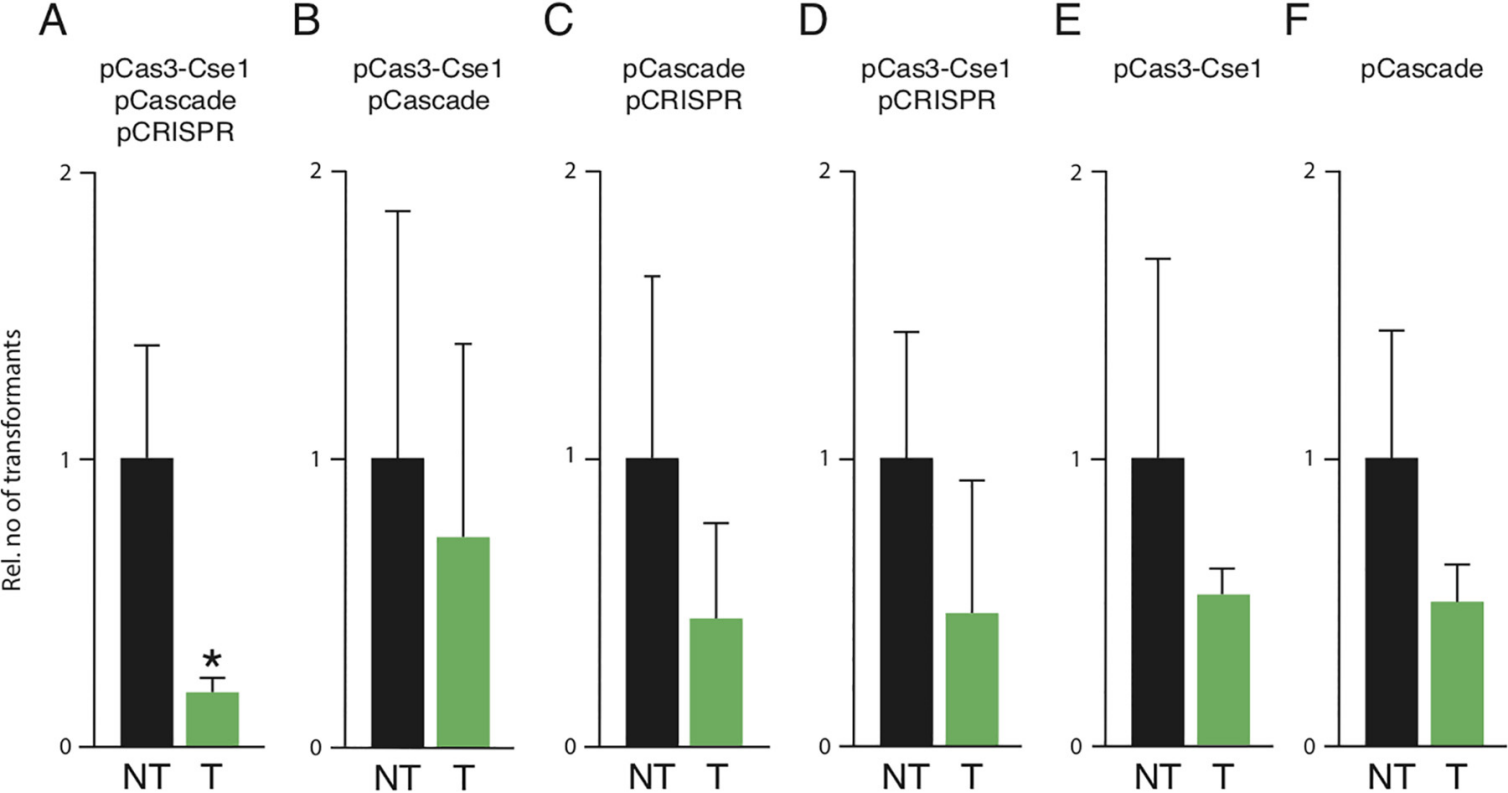
Analysis of CRISPR-Cas interference in S. cerevisiae with complete immune system (A) or when missing CRISPR (B), Cas3 (C), Cascade (D), Cascade and CRISPR (E), or Cas3 and CRISPR (F). Transformation efficiency was determined by comparing transformation efficiency of targeted (T) with nontargeted (NT) pTargetHigh plasmids. Data are a summary of multiple independent biological replicates (A, *n* = 4; B, 3; C, 3; D, 5; E, 3; F, 3) normalized so that the relative level of transformation by the nontarget plasmid is equal in the different panels. Error bars indicate one standard deviation. A statistically significant reduction of transformation by T compared with NT as determined by the Kruskal-Wallis chi-square test is indicated by an asterisk (*P* ≤ 0.05).

The functionality of Cascade-Cas3 interference was assessed by comparing transformation efficiency of a plasmid targeted by the CRISPR-Cas system with that of the same plasmid lacking the target fragment. Transformations were done routinely at 240 min after the induction of *cas* and CRISPR expression. Using pCas3, pCascade, and pCRISPR in S. cerevisiae W303, a 47% decrease in transformation efficiency of pTargetHigh was observed (Fig. 2B). Tests with a 150-min induction produced in similar results (see Fig. S1 in the supplemental material). An analysis of interference with or without induction demonstrated interference under both conditions (see Fig. S2 in the supplemental material), indicating that basal expression from the Gal promoter was sufficient to achieve activity. We used a Cas3-Cse1 fusion construct to test if colocalization of Cascade and Cas3 improved interference, and we indeed observed further (73.5%) reduction in the transformation efficiency of pTargetHigh in S. cerevisiae W303 (Fig. 2C). Control experiments demonstrated that transformation efficiency of the targeted plasmid was on average lower than that of the nontargeted plasmid also when interference could not occur, i.e., when Cas3-Cse1, Cascade, and/or CRISPR was absent; however, the difference in transformation efficiency was largest, and only statistically significant, in interference-competent cells (Fig. 3, primary data available in Table S2 in the supplemental material). To ensure that activity was not limited to a single experimental condition, we tested two different versions of our Cascade-Cas3 system on a different target, namely, a plasmid with a low copy number carrying different target sequences. Expression of pCRISPR+Cas3 or pCRISPR+Cas3-Cse1 together with pCascade in S. cerevisiae BY418 resulted in 88.6% and 88.7% reduction in transformation efficiency of pTargetLow, respectively (Fig. 2D and E).

10.1128/msphere.00038-22.1 FIG S1 Analysis of CRISPR-Cas interference in S. cerevisiae with 150 min (A) or 240 min (B) induction of pCas3, pCascade, and pCRISPR by determining transformation efficiency with targeted (T) or nontargeted plasmids (NT). Data are a summary of three independent biological replicates normalized so that the relative level of transformation by the nontarget plasmid is equal in the different panels. Error bars indicate one standard deviation. Download FIG S1, TIF file, 0.06 MB. Copyright © 2022 Bindal et al. 2022 Bindal et al. https://creativecommons.org/licenses/by/4.0/ This content is distributed under the terms of the Creative Commons Attribution 4.0 International license.

10.1128/msphere.00038-22.2 FIG S2 Analysis of the effect of CRISPR-Cas induction on interference with plasmids in different S. cerevisiae strains with the indicated CRISPR-Cas expression system. Transformation was performed with either a plasmid containing a target sequence (T), or the nontargeted parent plasmid (NT). Data in each bar are a summary of three independent biological replicates normalized so that the relative level of transformation by the nontarget plasmid is equal in the different panels. Error bars indicate one standard deviation. A statistically significant reduction of transformation by T compared with NT as determined by Mann-Whitney U test is indicated by an asterisk (*P* ≤ 0.05). Download FIG S2, TIF file, 0.2 MB. Copyright © 2022 Bindal et al. 2022 Bindal et al. https://creativecommons.org/licenses/by/4.0/ This content is distributed under the terms of the Creative Commons Attribution 4.0 International license.

10.1128/msphere.00038-22.6 TABLE S2 Transformation data for interference-active and control S. cerevisiae strains. Download Table S2, DOCX file, 0.01 MB. Copyright © 2022 Bindal et al. 2022 Bindal et al. https://creativecommons.org/licenses/by/4.0/ This content is distributed under the terms of the Creative Commons Attribution 4.0 International license.

### Effect of interference.

Interference assays demonstrated that CRISPR-Cas targeting reduced transformation efficiency but transformants were still observed on selective plates. These transformants could be carrying target plasmids that had not been cleaved by Cascade-Cas3, plasmids that had been cleaved but subsequently been repaired, or plasmids that had acquired escape mutations in the region targeted by CRISPR-Cas. NHEJ repair or the presence of different escape mutations would result in target sequence heterogeneity in the population of transformed target plasmids. To determine sequence heterogeneity in the transformed target plasmids, we performed the Surveyor assay, in which Surveyor nuclease cleaves the heterogeneous DNA with high specificity at the 3′ side of any mismatch site in both DNA strands, including all base substitutions and insertion/deletions. This mismatch cleavage assay has been used frequently to detect substitutions and indels in target DNA cut by Cas nuclease (12, 21). This assay is based on the principle that DNA with mismatched bases formed by denaturing and reannealing DNA strands with sequence differences would be detected and cleaved by the surveyor nuclease.

The target region of pTargetHigh was amplified by PCR and analyzed by the Surveyor assay. Sequence heterogeneity at the J3 target site would result in two cleavage fragments, which are both about 160 to 170 bp. No such cleavage products could be detected in strains expressing pCRISPR, pCascade, and pCas3-Cse1, similar to interference-negative-control strains (see Fig. S3 in the supplemental material). Moreover, we amplified and sequenced the target region in 20 independent survivor transformants with target-specific primers (DR003 and DR004). No heterogeneity in PAM and the protospacer sequence was observed (see Fig. S4 in the supplemental material). Overall, this finding suggests that the plasmid in transformants did not contain any escape mutations in the J region.

10.1128/msphere.00038-22.3 FIG S3 Capillary electrophoresis analysis of the Surveyor assay for the detection of mutations induced by the CRISPR-Cas system. S. cerevisiae with different plasmids was transformed with pTargetHigh. The experiments were done with or without a galactose inducer, as indicated. Lanes 2 to 4 contain an analysis of 632-bp control DNA with a C-G heteroduplex or C-C homoduplex at position 432. The sizes of the original DNA and expected Surveyor cleavage products of control DNA (red) and target DNA (black) are indicated. Download FIG S3, TIF file, 0.3 MB. Copyright © 2022 Bindal et al. 2022 Bindal et al. https://creativecommons.org/licenses/by/4.0/ This content is distributed under the terms of the Creative Commons Attribution 4.0 International license.

10.1128/msphere.00038-22.4 FIG S4 Multiple sequence alignment shows the target J fragment sequence aligned with sequences obtained from 20 survivor transformants. The protospacer is highlighted in red, and PAM is highlighted in blue. The sequence alignment is used to determine escape mutation or any repair in the target plasmid used for CRISPR interference assays. Download FIG S4, TIF file, 0.4 MB. Copyright © 2022 Bindal et al. 2022 Bindal et al. https://creativecommons.org/licenses/by/4.0/ This content is distributed under the terms of the Creative Commons Attribution 4.0 International license.

## DISCUSSION

We demonstrate successful reconstitution of bacterial type I-E CRISPR-Cas interference in the eukaryote S. cerevisiae. We detect accurate processing of crRNA by Northern blotting and, importantly, a reduction of target plasmid transformation rate compared with a nontargeted control plasmid. We could not detect sequence heterogeneity in target plasmids recovered from transformed cells. We do not rule out that rare events of sequence heterogeneity are present, but the results demonstrate that the level of interference observed was due to the activity of Cascade-Cas3 and not by various levels of escape mutations in the target. The lack of observable sequence heterogeneity also demonstrates that NHEJ repair of the CRISPR-Cas-induced damage is not occurring at a high level, although this result was expected as NHEJ repair is rare in S. cerevisiae (22). Our results suggest that the programmable processive DNA degradation by the type I-E CRISPR-Cas system can be restored in eukaryotic cells.

In our initial design, Cascade, Cas3, and pre-crRNA are expressed from different plasmids and resulted in 50% reduction in plasmid transformation rate. To improve activity, we developed an improved system. Coexpression of a Cas3-Cse1 fusion results in a subpopulation of Cascade complexes permanently colocalized with Cas3, which reduced transformation by 73.5%, a level similar to when J3 crRNAs are used against phage Lambda in E. coli expressing the LeuO activator (20). We hypothesize that this fusion improves the kinetics of target degradation, as the factors do not need to find each other; alternatively, Cas3 or Cas1 may be a limiting factor in the process and the addition of the fusion protein boosts the activity. Furthermore, to ensure that the effect was not limited to a specific type of target plasmid, we also tested activity against a low copy target plasmid with different target sequences and observed that only about 1-in-10 plasmids successfully established themselves in the cell compared with a nontargeted control.

Different crRNAs are known to result in different levels of CRISPR-Cas system interference (7, 23, 24), so other spacer sequences may further increase the efficiency of target degradation in yeast. An analysis of Cascade and Cas3 proteins with NucPred (25) and NLSdb (26) did not show the presence of nuclear localization signals (NLS)-like motifs (data not shown). We also speculate that the Cascade and Cas3 in the absence of NLS locate to the cytoplasm, thus allowing only a narrow window of opportunity to destroy their target plasmids while they are enroute to the nucleus. This narrow window would cause a reduction in target access and interference compared with bacteria and archaea, which typically lack an access-restricting membrane around their nucleoid. Other factors that may further improve interference in S. cerevisiae are increased expression of Cas protein and pre-crRNA, codon optimization, prevention of detrimental posttranslational modification of Cas proteins, and coexpression of E. coli host factors that may assist interference.

The large type I-E system is more difficult to establish heterologously than the more compact class 2 systems (3, 23, 27). However, complete target clearance by type I systems is a distinct difference to the inactivation of MGEs by mutagenesis that is achieved by, e.g., class 2 CRISPR-Cas systems. To inactivate by mutation, a thorough understanding of the biology of MGEs is required to find a key component to target. Also, compared with Cas9-based systems, type I systems can target multiple sequences by adding different spacer-repeat units to the CRISPR (23, 28) In conventional Cas9 technology, multiple single guide RNA genes are required to achieve such multiplexing (29, 30).

Another benefit of our establishment of a type I-E system in S. cerevisiae is that it functions as a platform for testing our current understanding of the system in a nonnative background. Such a platform would allow an evaluation of proteins and other factors for their role in the immune system.

Finally, while we demonstrate formation of mature crRNA and interference in a Saccharomyces cerevisiae, further development could reconstitute the entire adaptive capability of the system.

## MATERIALS AND METHODS

### Strains and culture media.

All cloning work was done in E. coli strain Top10. E. coli was cultured in lysogeny broth (LB) with aeration at 37°C. When necessary, the medium was supplemented with kanamycin (50 μg/mL), ampicillin (100 μg/mL), streptomycin (50 μg/mL), tetracycline (25 μg/mL), and chloramphenicol (15 μg/mL). For experiments in yeast, S. cerevisiae W303 and BY418 strains were used. S. cerevisiae BY418 has a full deletion of chromosomal copies of genes used for selection (his3, leu2, trp1, and ura3), and in W303, they are inactivated by point mutations. Yeast cells were cultured in synthetic complete (SC) medium (0.34% yeast nitrogen base, 0.5% ammonium sulfate, 2% raffinose, and synthetic complete mixture (Kaiser) quadruple drop-out mix [-His, -Leu, -Trp, -Ura, Formedium, UK] at the rate of 1,400 mg/L of medium [pH 5.6]). For certain induction experiments, SC medium with 1% raffinose was used. When necessary, the medium was supplemented with adenine sulfate (80 μg/mL), uracil (20 μg/mL), tryptophan (40 μg/mL), histidine (20 μg/mL), and leucine (60 μg/mL). A total of 1.5% agar was included in the medium to prepare the solid medium. Yeast strains were grown with aeration at 32°C. A total of 2% galactose was added to the medium for induction of the Gal promoter as required. For a complete list of strains, see Table S3 in the supplemental material.

10.1128/msphere.00038-22.7 TABLE S3 List of strains. Download Table S3, DOCX file, 0.01 MB. Copyright © 2022 Bindal et al. 2022 Bindal et al. https://creativecommons.org/licenses/by/4.0/ This content is distributed under the terms of the Creative Commons Attribution 4.0 International license.

### Construction of a vector expressing the Cascade complex.

A Gal1-10 promoter cassette from pRS425Gal was cloned between EcoRI/BamHI sites of pBlueScript II SK (+) resulting in pUDM101. All *cas* genes were amplified by colony PCR from E. coli MG1655; see Table S4 in the supplemental material for a list of primers. The *cse1* and *cse2* genes were cloned serially into the pUDM101 XbaI and EcoRI sites, respectively, to generate pUDM107. Similarly, *cas7* and *cas6e* were cloned serially in pRS425Gal BamHI and SalI sites, respectively, to generate pUDM108. The four genes were combined by subcloning a HindIII-NotI blunt fragment from pUDM107 into the blunted SacI site of pUDM108, thereby generating pUDM109. The *cas5e* gene was cloned into a pRS425Gal vector to generate pUDM110. A CYC1 terminator cassette was PCR amplified from pYES2 and subcloned into the PstI site of pBlueScript II SK (+) to generate pUDM102. A CYC1-*cas5e*-CYC1 cassette was constructed by serially cloning a EcoRV-SmaI fragment from pUDM102 into the SalI and SpeI sites (blunted with T4 DNA polymerase), respectively, of pUDM110 to generate pUDM315. Using XhoI and NotI, this cassette was excised from pUDM315, blunted with T4 DNA polymerase, and subcloned into the NotI site (blunted with T4 DNA polymerase) of pUDM109 to generate the final Cascade-expressing construct pCascade. See Table S5 in the supplemental material for a list of plasmids used.

10.1128/msphere.00038-22.8 TABLE S4 List of primers. Download Table S4, DOCX file, 0.01 MB. Copyright © 2022 Bindal et al. 2022 Bindal et al. https://creativecommons.org/licenses/by/4.0/ This content is distributed under the terms of the Creative Commons Attribution 4.0 International license.

10.1128/msphere.00038-22.9 TABLE S5 List of plasmids. Download Table S5, DOCX file, 0.02 MB. Copyright © 2022 Bindal et al. 2022 Bindal et al. https://creativecommons.org/licenses/by/4.0/ This content is distributed under the terms of the Creative Commons Attribution 4.0 International license.

### Construction of vectors expressing Cas3 or Cas3-Cse1 fusion.

The *cas3* gene was PCR amplified from MG1655 to introduce flanking EcoRI sites and cloned into the EcoRI site of pRS423Gal resulting in the Cas3-expressing plasmid pCas3. A *cas3*-*cse1* fusion fragment was amplified from pWUR657 and cloned between the SpeI and NotI sites of pRS423Gal to generate pCas3-Cse1.

### Construction of vectors expressing CRISPR RNA.

For the expression of crRNA, two CRISPR cassettes, namely, one containing 4xJ3 spacer (20) PCR amplified from pWUR630 and the other containing J1-J2 spacers (a synthetic array from ThermoFisher Scientific Inc.), were cloned into BamHI-NotI sites of pRS424Gal_Cyc1 to place the CYC1 terminator directly downstream of the CRISPR array. All spacer sequences were selected by identifying defined canonical PAMs (ATG and AGG) for the type I-E system on the target J gene from bacteriophage Lambda (8, 9). The J1-J2 CRISPR, along with the flanking CYC1 terminator, was excised as a BamHI-SacI fragment and cloned in pCas3 cut with the same sites to generate pCRISPR+Cas3. Additionally, the same BamHI-SacI fragment was blunted and cloned in the blunted SalI site of pCas3-Cse1 to generate pCRISPR+Cas3-Cse1.

For the expression crRNA in E. coli, a minimal CRISPR array with 54 nt of the leader, the J3 spacer, and two repeats was cloned into pZE12Luc (31) under the control of PLlacO-1. The plasmid was amplified using primers PLlacO-C and pZE-Xba, and the minimal CRISPR array was amplified from pWUR564 (20) using primers LA009 and LA013. The array was cloned by blunt-end ligation in the leader-end so that the first position of the partial leader corresponds to the transcription start of the PLlacO-1 and in the other end into the XbaI-site of pZE12Luc. See Table S1 for details of CRISPRs, targets, and cloned constructs.

### Construction of S. cerevisiae expressing the type I-E CRISPR-Cas system.

The vectors constructed as described above were transformed into the desired yeast strains as required. Transformation of S. cerevisiae was performed using the lithium acetate method (32).

### Construction of target vectors.

To construct pTargetHigh, the 350-bp region of Lambda J from pWUR610 (33) was cloned into the BamHI and HindIII sites of pRS426Gal. The cloning replaced the Gal-promoter fragment with the Lambda DNA fragment. A low copy target vector, pTargetLow, was constructed by PCR amplification of a region of Lambda J gene using pTargetHigh as the template with DR0015 and DR0016 primers and cloning the fragment in the NheI site of pPS1739 (See Table S1).

### Analysis of crRNA processing.

**(i) Sample preparation and RNA purification: yeast.** The S. cerevisiae W303 strain carrying pCascade, pCRISPR (4xJ3 spacer), and pCas3 was grown to stationary phase, diluted in fresh SC medium, and grown with aeration at 32°C to an optical density at 600 nm (OD_600_) value of 0.3. Galactose was then added to induce the CRISPR-Cas system. A total of 10 mL culture was harvested by centrifugation at 2, 4, and 5 h after induction. Total RNA was isolated using the hot phenol method (34).

### (ii) Sample preparation and RNA purification: bacteria.

An overnight culture of E. coli BL21AI harboring pWUR397 (Cas3), pWUR400 (Cascade), and pLA002 (minimal J3 CRISPR array) was diluted and grown to an OD_600_ of ≈0.3. Cas protein and crRNA expression were induced by addition of 0.2% arabinose and 1 mM isopropyl-β-d-thiogalactopyranoside (IPTG). After 30 min, a 5-mL sample was taken and mixed with 1-mL stop solution (5% phenol and 95% ethanol) and pelleted at 4°C. RNA was isolated as described previously (35).

### (iii) Gel and blotting.

Samples were analyzed on a 10% denaturing polyacrylamide gel (10% polyacrylamide, 7 M urea, and 1× Tris-borate-EDTA [TBE]) in 1× TBE. Samples were mixed with loading dye (95% deionized formamide, 0.5 mM EDTA, 0.025% bromophenol blue-xylene cyanol, and 0.025% SDS) and boiled 3 min prior to loading. The same was done with the prelabeled pUC8 size marker. Next, 15 μg of total RNA was loaded for each sample. As a positive control for processed crRNA, total RNA purified from the E. coli BL21AI expressing pWUR397, pWUR400, and pLA002, was also loaded on the gel.

Electrotransfer to a Hybond N+ membrane (Amersham) was done at 4°C overnight at 200 mA. The membrane was UV cross-linked and prehydridized in Church buffer (0.25 M sodium phosphate buffer [pH 7.2], 1 mM EDTA, and 7% SDS) for 1 h at 42°C after which 0.5 μM radioactively labeled probe was added to the buffer. Hybridization was done overnight. The membrane was washed two times for 5 min each with 2× SSC (1× SSC is 0.15 M NaCl plus 0.015 M sodium citrate) and 0.1% SDS and exposed on the PharosFX-system (Bio-Rad).

The pUC8 size marker (Fermentas) was labeled radioactively using γ-^32^P-ATP (PerkinElmer) and polynucleotide kinase (PNK; Fermentas) in an exchange reaction according to the manufacturer’s instructions. The probe LA014 was labeled the same way in a forward reaction. Excess γ-^32^P-ATP was removed using the Illustra ProbeQuant G-50 Micro column (GE Healthcare) according to the manufacturer’s instructions.

### CRISPR-Cas activity assays.

CRISPR-Cas activity was assessed by plasmid interference assays. Yeast cultures were grown to stationary phase and diluted in fresh SC medium containing either 1% or 2% raffinose as the carbon source and grown with aeration at 32°C to an OD_600_ value of 0.2, followed by induction with galactose. A total of 150 to 240 min after induction. the cells were harvested by centrifugation. The cells were then transformed with 280 ng of target vectors or nontarget control vectors using the lithium acetate method (32) but excluding carrier DNA. Plasmid interference by the CRISPR-Cas system was measured by plating cultures transformed with the target vector or nontarget vector on selective media (SC medium lacking uracil) and comparing transformation efficiency (TE). TE was calculated by determining the number of CFU recovered per μg of DNA transformed. Experiments were performed with 3 to 5 biological replicates.

### Surveyor assay.

The Surveyor assay allows the detection of mutations and polymorphisms in DNA. If two strands of DNA form a heteroduplex due to the presence of single nucleotide polymorphisms (SNPs) or small insertions or deletions, the Surveyor nuclease recognizes the mismatch and cleaves the DNA. The cleaved DNA products can be detected by gel electrophoresis (36).

S. cerevisiae BY418 cells harboring pCascade, pCRISPR, and pCas3-Cse1 or negative controls lacking pCRISPR, pCascade, or both were grown in SC medium with or without the galactose inducer and transformed with pTargetHigh. The transformed cells were grown for 24 h in SC medium, pelleted by centrifugation, and grown again in equal volume of fresh medium for 24 h. A total of 1.5 mL of the culture was pelleted by centrifugation and resuspended in 500 μL of distilled water. The cells were lysed by heating at 98°C for 10 min, and 0.5 μL of the lysate was used as the template for PCR with primers DR003 and DR004 to amplify the target region. The PCR products were analyzed by electrophoresis on a 2% agarose gel and used for mutation detection with a Surveyor mutation detection kit (IDT) as per the manufacturer’s instructions. The result of the Surveyor assay was analyzed by using the Qiagen QIAxcel advanced gel electrophoresis system.
