# Supplementary material for: Type I-E CRISPR-Cas System as a Defense System in Saccharomyces cerevisiae
Source: mSphere. 2022 Apr 27;7(3):e00038-22. doi: 10.1128/msphere.00038-22 (PMC9241507; doi:10.1128/msphere.00038-22)
Supplement: TABLE S1 [file msphere.00038-22-s0005.docx]

**Table S1**: Sequences of constructs, CRISPRs and targets

| Name | Sequence | Remark |
| --- | --- | --- |
| 4×J3 CRISPR-spacer construct | CGAATTGAAGGAAGGCCGTCAAGGCCGCATGAGCTCCCATGGAAACAAAGAATTAGCTGATCTTTAATAATAAGGAAATGTTACATTAAGGTTGGTGGGTTGTTTTTATGGGAAAAAATGCTTTAAGAACAAATGTATACTTCTAGA**GAGTTCCCCGCGCCAGCGGGGATAAACCG**CCAGTGATAAGTGGAATGCCATGTGGGCTGTC**GAGTTCCCCGCGCCAGCGGGGATAAACCG**CCAGTGATAAGTGGAATGCCATGTGGGCTGTC**GAGTTCCCCGCGCCAGCGGGGATAAACCG**CCAGTGATAAGTGGAATGCCATGTGGGCTGTC**GAGTTCCCCGCGCCAGCGGGGATAAACCG**CCAGTGATAAGTGGAATGCCATGTGGGCTGTC**GAGTTCCCCGCGCCAGCGGGGATAAACCG**CAGCTCCCATTTTCAAACCCAGGTACCCTGGGCCTCATGGGCCTTCCTTTCACTGCC | The spacers matching J3 protospacer are highlighted in yellow. The CRISPR repeats are marked in bold. The leader sequence is marked in red. CRISPR construct (pCRISPR) used in interference assays in W303. |
| J1-2 CRISPR-spacer construct | GGATCCGTGGGTTGTTTTTATGGGAAAAAATGCTTTAAGAACAAATGTATACTTTTAGA**GAGTTCCCCGCGCCAGCGGGGATAAACCG**tcgagctgacggaggataacgccagcagactg**GAGTTCCCCGCGCCAGCGGGGATAAACCG**tcgcgggtattggcctcagcatggaggacacg**GAGTTCCCCGCGCCAGCGGGGATAAACCGGCGGCCG**C | The spacers matching J1 and J2 protospacers highlighted in magenta and cyan, respectively. The CRISPR repeats are marked in bold. The leader sequence is marked in red. CRISPR construct (pCRISPR) used in interference assays in BY418. |
| J region | tcgggcgagcgatgatgcggaaggttacctggattttttcaaaggcaagataaccgaatcccatctcggcaaggagctgctggaaaaagtcgagctgacggaggataacgccagcagactggaggagttttcgaaagagtggaaggatgccagtgataagtggaatgccatgtgggctgtcaaaattgagcagaccaaagacggcaaacattatgtcgcgggtattggcctcagcatggaggacacggaggaaggcaaactgagccagtttctggttgccgccaatcgtatcgcatttattgacccggcaaacgggaatgaaacgccgatgt | Lambda J region used as target (pTargetHigh/ pTargetLow). The J1, J2 and J3 protospacers are highlighted in magenta, cyan and yellow, respectively. The PAM sequence is shown in green |
| Minimal CRISPR array | GGTGGGTTGTTTTTATGGGAAAAAATGCTTTAAGAACAAATGTATACTTCTAGA**GAGTTCCCCGCGCCAGCGGGGATAAACCG**CCAGTGATAAGTGGAATGCCATGTGGGCTGTC**GAGTTCCCCGCGCCAGCGGGGATAAACCG** | Minimal CRISPR array used in pLA002. The spacer matching J3 protospacer is highlighted in yellow. The CRISPR repeats are marked in bold. The leader sequence is marked in red. |
| Cascade construct | CAGCTGGCGAAAGGGGGATGTGCTGCAAGGCGATTAAGTTGGGTAACGCCAGGGTTTTCCCAGTCACGACGTTGTAAAACGACGGCCAGTGAGCGCGCGTAATACGACTCACTATAGGGCGAATTGGGTACCGGGCCCCCCCTCGAGGTCGACTGGGATTAAGGGGAAGCCAGGTCATTTTATTACACCTCAATCACAGTGGAGCCAAAGATAGCAAGCCACATCCCATCGATTTAGCTGGCCCAATACCTTGCTGTACAAGATCTATTAACGCTGGCGCGTCGTTGATGGTGAGCACACCTTCAAAGCAAACCGTTTGGATCTTTCCACTTTTACCATCACCAGAAAAATACTGTGGCCGTTCCGATATGGGATGCACATCTTCAACGCGCGCCGCATTGCCCAATTTACGTTGCAACCACGCGATTTGTTCTGCTTCTTTTATTAACGGAACCCGACAGCGTTTAATATTCCCTTTACTGTCCAGGCGCTTTTGATTGTCGAGAATAGTTTTGATCGGATTTGCCCGAAGCCGAAAATAGAGTGGAACACCAACCTGAAGTTGAAATTCAACCTGTTTAGTTTTAATGACTGTCGCAACGGCAGTTGAAACAGGCATTTGCGCTGACTGCAATAAAACATGACAGCCTTCTGGTGTGTTTCGCTTCTCAACATGAAAAAGAAAATCACGAGCAGCATCCGGTCTGTTTGGAAATAAATGCCATAATCCCTGGTGAAGTTGGTAAAGATCCCTGCTCCAGGCCCTGGCAATGATGACTTTACTGAGATACATCTATACCTCCTTTGTCGACGGTATCGATAAGCTTGATATCGAATTCCTTGAATTTTCAAAAATTCTTACTTTTTTTTTGGATGGACGCAAAGAAGTTTAATAATCATATTACATGGCATTACCACCATATACATATCCATATACATATCCATATCTAATCTTACTTATATGTTGTGGAAATGTAAAGAGCCCCATTATCTTAGCCTAAAAAAACCTTCTCTTTGGAACTTTCAGTAATACGCTTAACTGCTCATTGCTATATTGAAGTACGGATTAGAAGCCGCCGAGCGGGTGACAGCCCTCCGAAGGAAGACTCTCCTCCGTGCGTCCTCGTCTTCACCGGTCGCGTTCCTGAAACGCAGATGTGCCTCGCGCCGCACTGCTCCGAACAATAAAGATTCTACAATACTAGCTTTTATGGTTATGAAGAGGAAAAATTGGCAGTAACCTGGCCCCACAAACCTTCAAATGAACGAATCAAATTAACAACCATAGGATGATAATGCGATTAGTTTTTTAGCCTTATTTCTGGGGTAATTAATCAGCGAAGCGATGATTTTTGATCTATTAACAGATATATAAATGCAAAAACTGCATAACCACTTTAACTAATACTTTCAACATTTTCGGTTTGTATTACTTCTTATTCAAATGTAATAAAAGTATCAACAAAAAATTGTTAATATACCTCTATACTTTAACGTCAAGGAGAAAAAACCCCGGATCCGTAAGGAAACCTTTCTATGTCTAACTTTATCAATATTCATGTTCTGATCTCTCACAGCCCTTCATGTCTGAACCGCGACGATATGAACATGCAGAAAGACGCTATTTTCGGCGGCAAAAGACGAGTAAGAATTTCAAGTCAAAGCCTTAAACGTGCGATGCGTAAAAGTGGTTATTACGCACAAAATATTGGTGAATCCAGTCTCAGAACCATTCATCTTGCACAATTACGTGATGTTCTTCGGCAAAAACTTGGTGAACGTTTTGACCAAAAAATCATCGATAAGACATTAGCGCTGCTCTCCGGTAAATCAGTTGATGAAGCCGAAAAGATTTCTGCCGATGCGGTTACTCCCTGGGTTGTGGGAGAAATAGCCTGGTTCTGTGAGCAGGTTGCAAAAGCAGAGGCTGATAATCTGGATGATAAAAAGCTGCTCAAAGTTCTTAAGGAAGATATTGCCGCCATACGTGTGAATTTACAGCAGGGTGTTGATATTGCGCTTAGTGGAAGAATGGCAACCAGCGGCATGATGACTGAGTTGGGAAAAGTTGATGGTGCAATGTCCATTGCGCATGCGATCACTACTCATCAGGTTGATTCTGATATTGACTGGTTCACCGCTGTAGATGATTTACAGGAACAAGGTTCTGCACATCTGGGAACTCAGGAATTTTCATCGGGTGTTTTTTATCGTTATGCCAACATTAACCTCGCTCAACTTCAGGAAAATTTAGGTGGTGCCTCCAGGGAGCAGGCTCTGGAAATTGCAACCCATGTTGTTCATATGCTGGCAACAGAGGTCCCTGGAGCAAAACAGCGTACTTATGCCGCTTTTAACCCTGCGGATATGGTAATGGTTAATTTCTCCGATATGCCACTTTCTATGGCAAATGCTTTTGAAAAAGCGGTTAAAGCGAAAGATGGCTTTTTGCAACCGTCTATACAGGCGTTTAATCAATATTGGGATCGCGTTGCCAATGGATATGGTCTGAACGGAGCTGCTGCGCAATTCAGCTTATCTGATGTAGACCCAATTACTGCTCAAGTTAAACAAATGCCTACTTTAGAACAGTTAAAATCCTGGGTTCGTAATAATGGCGAGGCGTGAACATGAGATCTTATTTGATCTTGCGGCTTGCTGGGCCAATGCAAGCCTGGGGGCAGCCGACCTTTGAAGGAACGCGACCTACCGGAAGATTTCCGACCCGAAGCGGGTTATTAGGGCTACTCGGGGCTTGTCTTGGGATCCACTAGTTCTAGAGCGGCCAGAACTAGGGCTGCAGATTAAAGCCTTCGAGCGTCCCAAAACCTTCTCAAGCAAGGTTTTCAGTATAATGTTACATGCGTACACGCGTCTGTACAGAAAAAAAAGAAAAATTTGAAATATAAATAACGTTCTTAATACTAACATAACTATAAAAAAATAAATAGGGACCTAGACTTCAGGTTGTCTAACTCCTTCCTTTTCGGTTAGAGCGGATGTGGGGGGAGGGCGTGAATGTAAGCGTGACATAACTAACTGCAGGAATTCGATCTAGTGGATCCGGGGTTTTTTCTCCTTGACGTTAAAGTATAGAGGTATATTAACAATTTTTTGTTGATACTTTTATTACATTTGAATAAGAAGTAATACAAACCGAAAATGTTGAAAGTATTAGTTAAAGTGGTTATGCAGTTTTTGCATTTATATATCTGTTAATAGATCAAAAATCATCGCTTCGCTGATTAATTACCCCAGAAATAAGGCTAAAAAACTAATCGCATTATCATCCTATGGTTGTTAATTTGATTCGTTCATTTGAAGGTTTGTGGGGCCAGGTTACTGCCAATTTTTCCTCTTCATAACCATAAAAGCTAGTATTGTAGAATCTTTATTGTTCGGAGCAGTGCGGCGCGAGGCACATCTGCGTTTCAGGAACGCGACCGGTGAAGACGAGGACGCACGGAGGAGAGTCTTCCTTCGGAGGGCTGTCACCCGCTCGGCGGCTTCTAATCCGTACTTCAATATAGCAATGAGCAGTTAAGCGTATTACTGAAAGTTCCAAAGAGAAGGTTTTTTTAGGCTAAGATAATGGGGCTCTTTACATTTCCACAACATATAAGTAAGATTAGATATGGATATGTATATGGATATGTATATGGTGGTAATGCCATGTAATATGATTATTAAACTTCTTTGCGTCCATCCAAAAAAAAAGTAAGAATTTTTGAAAATTCAAGGAATTCGATATCAAGCTTGGCGAGGCGTGAACATGAGATCTTATTTGATCTTGCGGCTTGCTGGGCCAATGCAAGCCTGGGGGCAGCCGACCTTTGAAGGAACGCGACCTACCGGAAGATTTCCGACCCGAAGCGGGTTATTAGGGCTACTCGGGGCTTGTCTTGGGATCCAACGTGATGATACTTCTTCATTACAGGCGTTATCAGAGAGTGTGCAATTTGCAGTGCGCTGCGATGAACTCATTCTTGACGATCGTCGTGTGTCTGTAACGGGGTTGCGTGATTACCATACAGTCCTTGGAGCGCGAGAAGATTACCGTGGTTTGAAAAGTCATGAAACGATTCAAACATGGCGCGAATATTTATGTGATGCCTCCTTTACCGTCGCTCTCTGGTTAACACCCCATGCAACGATGGTTATCTCAGAACTTGAAAAAGCAGTATTAAAGCCTCGGTATACACCTTACCTGGGGCGGAGAAGTTGCCCACTAACACACCCGCTTTTTTTGGGGACATGTCAGGCATCGGATCCTCAGAAGGCGCTATTAAATTATGAGCCCGTTGGCGGCGATATATATAGTGAGGAATCAGTTACAGGGCATCATTTAAAATTTACGGCGCGCGACGAACCGATGATCACCTTGCCTCGACAATTTGCTTCCCGAGAATGGTATGTGATTAAAGGAGGTATGGATGTATCTCAGTAAAGTCATCATTGCCAGGGCCTGGAGCAGGGATCTTTACAAGCTTATCGATACCGTCGAGGGCTGCAGATTAAAGCCTTCGAGCGTCCCAAAACCTTCTCAAGCAAGGTTTTCAGTATAATGTTACATGCGTACACGCGTCTGTACAGAAAAAAAAGAAAAATTTGAAATATAAATAACGTTCTTAATACTAACATAACTATAAAAAAATAAATAGGGACCTAGACTTCAGGTTGTCTAACTCCTTCCTTTTCGGTTAGAGCGGATGTGGGGGGAGGGCGTGAATGTAAGCGTGACATAACTAACTGCAGGAATTCGATTCGACCTCGAGGCCGCCACCGCGGTGGGGCCGCTCTAGATATAAAGCCATTGCATCAATTTCATCAGCCATTTGATGGCCCTCCTTGCGGTTTTAACTCCCGTAAATGTTTGTATAGCGTGGCGCGGGCAAGCGCTAATGTGCTTATTAATTTAGGATGATGTGCATAGGGAGCTACAGATTGATTAAATAGCATTTCACACAATTGATGAAGTTTGTCTCGTAAATCAGCTATTACCTCATCAGCCTGGGAAAAATTAACATTCGCCAGTACATCGGGAATTAATAATTCACTCTGTCGATAGAAATGCCTTTCTGCAGTCTCATGAACAGAGACTCCGGCCCCTTTGAAGTCTTTATTTTTAAACCCTTCTGCAAAGGTATATAACGCCTTGCGTAAGGCTGTTTTATATCCCAAACCAACAGTCACTATTTCGTTTATCACATTGCCGTATTGTTGCCACCCCTGATTAAACATCAACACATCATGACGCCGTTCAAGAATAGATGCTTGATTATTACGATATCCCCCCATAATCAATTCAAGAGGACTTTGCGGCGCAATATTTCTGAATTGATTCACAACCGCCGCCACGCGATTTCCATTTTCATTTTGAATAATCTTATCTACCACAACTCGGCTGATTTGTGTCCATGATGGTGCGGAGGTGGTGAAAGCAAGAAATTTTTCCTCAACCTCCCCTTTCTTGACTGTTACCAGACAAGGGGAATGCGGATGGGGCCATAGCCCATTAACTGTAAAGGTAAATTTTTCCTTAAGAAAACCGGTATAACGCAAATTGCTTTCCTGTCCACAGCAAGAACATTTACCAATCCCAATGGGATCGCATAATTCAATATGCGCTGGTTGCCAGAATAGACCACGGACAAACCCAATTGACGAAGCAGGTATAGACTCATTGGACTTGATAGGTTTAATCCAGGTAGGTTGGTTTTCCGTATGTGATTCATTAGGAAATTGTTTTTGAAGACGAGGTAATGTGAGGACATTGAGTAACACCGTTGAACGAAGATCGATCCCACGTACGAACGTTGTTACAGGTGTTCCTCCACGTAAACCGCTTTTAAAACCACCACCAAAACCTGGTGCCTGATTCGCCTGGTTGAATAACGCAATCGCAGTGCATCCACCACATAATGCTTCACCCTGCCCCGGTTGATTGACAAATGCACAATTCGTCGCGCCGCTTACCCCAGCCAACAGTTTTTCCATTGGAGTCACATCATTTGCTTTGACACCTTTGGTCTGCATAAAGGGATGTTCTGCGTGATTAAGGTAGAACATATCTATCCACGGCGCGATGAGTTGTTGAAACTCATCTTCAGTGAGCGGATTCATTATGCGATGTCGAAATTCAACGTCATCTTTTGCCGGGGCGATAATTTGCCCAATGCAAACCAGCAGTGCTAAAGCGGCCAGTTCCATATCGTCACGGGGCAAACTTAATCGCCACTGATCTCTACTGCAGTATAGCGATTGCAGATTTATGATTTGGACTTTCCCCCCGTTTCGCGGGCGTACAGGGATCCAGTTATCAATAAGCAAATTCATTTGTTCTCCTTCGTATGCTTCTAGAACTAGTGGATCCGGGGTTTTTTCTCCTTGACGTTAAAGTATAGAGGTATATTAACAATTTTTTGTTGATACTTTTATTACATTTGAATAAGAAGTAATACAAACCGAAAATGTTGAAAGTATTAGTTAAAGTGGTTATGCAGTTTTTGCATTTATATATCTGTTAATAGATCAAAAATCATCGCTTCGCTGATTAATTACCCCAGAAATAAGGCTAAAAAACTAATCGCATTATCATCCTATGGTTGTTAATTTGATTCGTTCATTTGAAGGTTTGTGGGGCCAGGTTACTGCCAATTTTTCCTCTTCATAACCATAAAAGCTAGTATTGTAGAATCTTTATTGTTCGGAGCAGTGCGGCGCGAGGCACATCTGCGTTTCAGGAACGCGACCGGTGAAGACGAGGACGCACGGAGGAGAGTCTTCCTTCGGAGGGCTGTCACCCGCTCGGCGGCTTCTAATCCGTACTTCAATATAGCATACTCGTCAATTCGCATAATGACTTTCAAGGTTTCTCTTCCAAAAAAATCCGATTCTATTACCCCGAGAAATGTAAAGGTGTTGTATATTCATTCTAATCTATACCTATACATATACCTAATGTATATGGTGGTAATGCCATGTAATATGATTATTAAACTTCTTTGCGTCCATCCAAAATTTTTCATTCTTAAAAACTTTTAAGTTCCTTAAGTTTGGCGTTCCTCCCGGTAGTTTACCCTGATGAAATTGATGCAATGGCTTTATATCGAGCCTGGCAACAACTGGATAATGGATCATCACGCGTTTAATCTGCACAAAGTCTTGGACTACTTAATGCGCTATAGGGACGCAAAATATGGCTGGTGCAACCTTTTGGTTGGGAAAACCCACGTCACCAGCAGGCTCTTTTGCGCATGGACAAAACGGACTCGCGTCCTTTCTTACAGTAGGCTGTAGTCCTGTTTTTTAGCCTCGTTTCAACAGGTATCTCGTTGGGAAGAGCTTTAGCCAATAGTGGAAGAATTAACGAGCGCCGTAAGAAAGTTAATTAAGCCCGACTGTCTTGTCGGCTATACCAGGTCAATGCAGCTAATGAATCTCACGCCGAACCCGTACTTGACTGGCCATTAATGGCCAGGATGTTGACCTGGTGGGGAATCGCGCTTGCGGTCGTTGAAGACCTTCTAAAACATAACTGGTGTTTGTTTTTACGCATTCGAAACCTTTCTATGTCTAACTTTATCAGAATTCGATATCAAGCTCCAGCTTTTGTTCCCT | *cas* genes (*cse6e-cas7*-*cas5e*-*cse1-cse2*) cloned in pCascade |
| Cas3 construct | ATGGAACCTTTTAAATATATATGCCATTACTGGGGAAAATCCTCAAAAAGCTTGACGAAAGGAAATGATATTCATCTGTTAATTTATCATTGCCTTGATGTTGCTGCTGTTGCAGATTGCTGGTGGGATCAATCAGTCGTACTGCAAAATACTTTTTGCCGAAATGAAATGCTATCAAAACAGAGGGTGAAGGCCTGGCTGTTATTTTTCATTGCTCTTCATGATATTGGAAAGTTTGATATACGATTCCAATATAAATCAGCAGAAAGTTGGCTGAAATTAAATCCTGCAACGCCATCACTTAATGGTCCATCAACACAAATGTGCCGTAAATTTAATCATGGTGCAGCCGGTCTGTATTGGTTTAACCAGGATTCACTTTCAGAGCAATCTCTCGGGGATTTTTTCAGTTTTTTTGATGCCGCTCCTCATCCTTATGAGTCCTGGTTTCCATGGGTAGAGGCCGTTACAGGACATCATGGTTTTATATTACATTCCCAGGATCAAGATAAGTCGCGTTGGGAAATGCCAGCTTCTCTGGCATCTTATGCTGCGCAAGATAAACAGGCTCGTGAGGAGTGGATATCTGTACTGGAAGCATTATTTTTAACGCCAGCGGGGTTATCTATAAACGATATACCACCTGATTGTTCATCACTGTTAGCAGGTTTTTGCTCGCTTGCTGACTGGTTAGGCTCCTGGACTACAACGAATACCTTTCTGTTTAATGAGGATGCGCCTTCCGACATAAATGCTCTGAGAACGTATTTCCAGGACCGACAGCAGGATGCGAGCCGGGTATTGGAGTTGAGTGGACTTGTATCAAATAAGCGATGTTATGAAGGTGTTCATGCACTACTGGACAATGGCTATCAACCCAGACAATTACAGGTGTTAGTTGATGCTCTTCCAGTAGCTCCCGGGCTGACGGTAATAGAGGCACCTACAGGCTCCGGTAAAACGGAAACAGCGCTGGCCTATGCTTGGAAACTTATTGATCAACAAATTGCGGATAGTGTTATTTTTGCCCTCCCAACACAAGCTACCGCGAATGCTATGCTTACGAGAATGGAAGCGAGCGCGAGCCACTTATTTTCATCCCCAAATCTTATTCTTGCTCATGGCAATTCACGGTTTAACCACCTCTTTCAATCAATAAAATCACGCGCGATTACTGAACAGGGGCAAGAAGAAGCGTGGGTTCAGTGTTGTCAGTGGTTGTCACAAAGCAATAAGAAAGTGTTTCTTGGGCAAATCGGCGTTTGCACGATTGATCAGGTGTTGATATCGGTATTGCCAGTTAAACACCGCTTTATCCGTGGTTTGGGAATTGGTCGAAGTGTTTTAATTGTTGATGAAGTTCATGCTTACGACACCTATATGAACGGCTTGCTGGAGGCAGTGCTCAAGGCTCAGGCTGATGTGGGAGGGAGTGTTATTCTTCTTTCCGCAACCCTACCAATGAAACAAAAACAGAAACTTCTGGATACTTATGGTCTGCATACAGATCCAGTGGAAAATAACTCCGCATATCCACTCATTAACTGGCGAGGTGTGAATGGTGCGCAACGTTTTGATCTGCTAGCTCATCCAGAACAACTCCCGCCCCGCTTTTCGATTCAGCCAGAACCTATTTGTTTAGCTGACATGTTACCTGACCTTACGATGTTAGAGCGAATGATCGCAGCGGCAAACGCGGGTGCACAGGTCTGTCTTATTTGCAATTTGGTTGACGTTGCACAAGTATGCTACCAACGGCTAAAGGAGCTAAATAACACGCAAGTAGATATAGATTTGTTTCATGCGCGCTTTACGCTGAACGATCGTCGTGAAAAAGAGAATCGAGTTATTAGCAATTTCGGCAAAAATGGGAAGCGAAATGTTGGACGGATACTTGTCGCAACCCAGGTCGTGGAACAATCACTCGACGTTGATTTTGATTGGTTAATTACTCAGCATTGTCCTGCAGATTTGCTTTTCCAACGATTGGGCCGTTTACATCGCCATCATCGCAAATATCGTCCCGCTGGTTTTGAGATTCCTGTTGCCACCATTTTGCTGCCTGATGGCGAGGGTTACGGACGACATGAGCATATTTATAGCAACGTTAGAGTCATGTGGCGGACGCAGCAACATATTGAGGAGCTTAATGGAGCATCCTTATTTTTCCCTGATGCTTACCGGCAATGGCTGGATAGCATTTACGATGATGCGGAAATGGATGAGCCAGAATGGGTCGGCAATGGCATGGATAAATTTGAAAGCGCCGAGTGTGAAAAAAGGTTCAAGGCTCGCAAGGTCCTGCAGTGGGCTGAAGAATATAGCTTGCAGGATAACGATGAAACCATTCTTGCGGTAACGAGGGATGGGGAAATGAGCCTGCCATTATTGCCTTATGTACAAACGTCTTCAGGTAAACAACTGCTCGATGGCCAGGTCTACGAGGACCTAAGTCATGAACAGCAGTATGAGGCGCTTGCACTTAATCGCGTCAATGTACCCTTCACCTGGAAACGTAGTTTTTCTGAAGTAGTAGATGAAGATGGGTTACTTTGGCTGGAAGGGAAACAGAATCTGGATGGATGGGTCTGGCAGGGTAACAGTATTGTTATTACCTATACAGGGGATGAAGGGATGACCAGAGTCATCCCTGCAAATCCCAAATAA | *cas3* gene insert in pCas3 |
| Cas3-Cse1 construct | CCatgGAACCTTTTAAATATATATGCCATTACTGGGGAAAATCCTCAAAAAGCTTGACGAAAGGAAATGATATTCATCTGTTAATTTATCATTGCCTTGATGTTGCTGCTGTTGCAGATT  GCTGGTGGGATCAATCAGTCGTACTGCAAAATACTTTTTGCCGAAATGAAATGCTATCAAAACAGAGGGTGAAGGCCTGGCTGTTATTTTTCATTGCTCTTCATGATATTGGAAAGTTTG  ATATACGATTCCAATATAAATCAGCAGAAAGTTGGCTGAAATTAAATCCTGCAACGCCATCACTTAATGGTCCATCAACACAAATGTGCCGTAAATTTAATCATGGTGCAGCCGGTCTGT  ATTGGTTTAACCAGGATTCACTTTCAGAGCAATCTCTCGGGGATTTTTTCAGTTTTTTTGATGCCGCTCCTCATCCTTATGAGTCCTGGTTTCCATGGGTAGAGGCCGTTACAGGACATC  ATGGTTTTATATTACATTCCCAGGATCAAGATAAGTCGCGTTGGGAAATGCCAGCTTCTCTGGCATCTTATGCTGCGCAAGATAAACAGGCTCGTGAGGAGTGGATATCTGTACTGGAAG  CATTATTTTTAACGCCAGCGGGGTTATCTATAAACGATATACCACCTGATTGTTCATCACTGTTAGCAGGTTTTTGCTCGCTTGCTGACTGGTTAGGCTCCTGGACTACAACGAATACCT  TTCTGTTTAATGAGGATGCGCCTTCCGACATAAATGCTCTGAGAACGTATTTCCAGGACCGACAGCAGGATGCGAGCCGGGTATTGGAGTTGAGTGGACTTGTATCAAATAAGCGATGTT  ATGAAGGTGTTCATGCACTACTGGACAATGGCTATCAACCCAGACAATTACAGGTGTTAGTTGATGCTCTTCCAGTAGCTCCCGGGCTGACGGTAATAGAGGCACCTACAGGCTCCGGTA  AAACGGAAACAGCGCTGGCCTATGCTTGGAAACTTATTGATCAACAAATTGCGGATAGTGTTATTTTTGCCCTCCCAACACAAGCTACCGCGAATGCTATGCTTACGAGAATGGAAGCGA  GCGCGAGCCACTTATTTTCATCCCCAAATCTTATTCTTGCTCATGGCAATTCACGGTTTAACCACCTCTTTCAATCAATAAAATCACGCGCGATTACTGAACAGGGGCAAGAAGAAGCGT  GGGTTCAGTGTTGTCAGTGGTTGTCACAAAGCAATAAGAAAGTGTTTCTTGGGCAAATCGGCGTTTGCACGATTGATCAGGTGTTGATATCGGTATTGCCAGTTAAACACCGCTTTATCC  GTGGTTTGGGAATTGGTCGAAGTGTTTTAATTGTTGATGAAGTTCATGCTTACGACACCTATATGAACGGCTTGCTGGAGGCAGTGCTCAAGGCTCAGGCTGATGTGGGAGGGAGTGTTA  TTCTTCTTTCCGCAACCCTACCAATGAAACAAAAACAGAAACTTCTGGATACTTATGGTCTGCATACAGATCCAGTGGAAAATAACTCCGCATATCCACTCATTAACTGGCGAGGTGTGA  ATGGTGCGCAACGTTTTGATCTGCTAGCTCATCCAGAACAACTCCCGCCCCGCTTTTCGATTCAGCCAGAACCTATTTGTTTAGCTGACATGTTACCTGACCTTACGATGTTAGAGCGAA  TGATCGCAGCGGCAAACGCGGGTGCACAGGTCTGTCTTATTTGCAATTTGGTTGACGTTGCACAAGTATGCTACCAACGGCTAAAGGAGCTAAATAACACGCAAGTAGATATAGATTTGT  TTCATGCGCGCTTTACGCTGAACGATCGTCGTGAAAAAGAGAATCGAGTTATTAGCAATTTCGGCAAAAATGGGAAGCGAAATGTTGGACGGATACTTGTCGCAACCCAGGTCGTGGAAC  AATCACTCGACGTTGATTTTGATTGGTTAATTACTCAGCATTGTCCTGCAGATTTGCTTTTCCAACGATTGGGCCGTTTACATCGCCATCATCGCAAATATCGTCCCGCTGGTTTTGAGA  TTCCTGTTGCCACCATTTTGCTGCCTGATGGCGAGGGTTACGGACGACATGAGCATATTTATAGCAACGTTAGAGTCATGTGGCGGACGCAGCAACATATTGAGGAGCTTAATGGAGCAT  CCTTATTTTTCCCTGATGCTTACCGGCAATGGCTGGATAGCATTTACGATGATGCGGAAATGGATGAGCCAGAATGGGTCGGCAATGGCATGGATAAATTTGAAAGCGCCGAGTGTGAAA  AAAGGTTCAAGGCTCGCAAGGTCCTGCAGTGGGCTGAAGAATATAGCTTGCAGGATAACGATGAAACCATTCTTGCGGTAACGAGGGATGGGGAAATGAGCCTGCCATTATTGCCTTATG  TACAAACGTCTTCAGGTAAACAACTGCTCGATGGCCAGGTCTACGAGGACCTAAGTCATGAACAGCAGTATGAGGCGCTTGCACTTAATCGCGTCAATGTACCCTTCACCTGGAAACGTA  GTTTTTCTGAAGTAGTAGATGAAGATGGGTTACTTTGGCTGGAAGGGAAACAGAATCTGGATGGATGGGTCTGGCAGGGTAACAGTATTGTTATTACCTATACAGGGGATGAAGGGATGA  CCAGAGTCATCCCTGCAAATCCCAAAGCGGATCCCACCAACCGCGCGAAAGGCCTGGAAGCGGTGAGCGTGGCGAGCatgAATTTGCTTATTGATAACTGGATTCCTGTACGCCCGCGAA  ACGGGGGGAAAGTCCAAATCATAAATCTGCAATCGCTATACTGCAGTAGAGATCAGTGGCGATTAAGTTTGCCCCGTGACGATATGGAACTGGCCGCTTTAGCACTGCTGGTTTGCATTG  GGCAAATTATCGCCCCGGCAAAAGATGACGTTGAATTTCGACATCGCATAATGAATCCGCTCACTGAAGATGAGTTTCAACAACTCATCGCGCCGTGGATAGATATGTTCTACCTTAATC  ACGCAGAACATCCCTTTATGCAGACCAAAGGTGTCAAAGCAAATGATGTGACTCCAATGGAAAAACTGTTGGCTGGGGTAAGCGGCGCGACGAATTGTGCATTTGTCAATCAACCGGGGC  AGGGTGAAGCATTATGTGGTGGATGCACTGCGATTGCGTTATTCAACCAGGCGAATCAGGCACCAGGTTTTGGTGGTGGTTTTAAAAGCGGTTTACGTGGAGGAACACCTGTAACAACGT  TCGTACGTGGGATCGATCTTCGTTCAACGGTGTTACTCAATGTCCTCACATTACCTCGTCTTCAAAAACAATTTCCTAATGAATCACATACGGAAAACCAACCTACCTGGATTAAACCTA  TCAAGTCCAATGAGTCTATACCTGCTTCGTCAATTGGGTTTGTCCGTGGTCTATTCTGGCAACCAGCGCATATTGAATTATGCGATCCCATTGGGATTGGTAAATGTTCTTGCTGTGGAC  AGGAAAGCAATTTGCGTTATACCGGTTTTCTTAAGGAAAAATTTACCTTTACAGTTAATGGGCTATGGCCCCATCCGCATTCCCCTTGTCTGGTAACAGTCAAGAAAGGGGAGGTTGAGG  AAAAATTTCTTGCTTTCACCACCTCCGCACCATCATGGACACAAATCAGCCGAGTTGTGGTAGATAAGATTATTCAAAATGAAAATGGAAATCGCGTGGCGGCGGTTGTGAATCAATTCA  GAAATATTGCGCCGCAAAGTCCTCTTGAATTGATTATGGGGGGATATCGTAATAATCAAGCATCTATTCTTGAACGGCGTCATGATGTGTTGATGTTTAATCAGGGGTGGCAACAATACG  GCAATGTGATAAACGAAATAGTGACTGTTGGTTTGGGATATAAAACAGCCTTACGCAAGGCGTTATATACCTTTGCAGAAGGGTTTAAAAATAAAGACTTCAAAGGGGCCGGAGTCTCTG  TTCATGAGACTGCAGAAAGGCATTTCTATCGACAGAGTGAATTATTAATTCCCGATGTACTGGCGAATGTTAATTTTTCCCAGGCTGATGAGGTAATAGCTGATTTACGAGACAAACTTC  ATCAATTGTGTGAAATGCTATTTAATCAATCTGTAGCTCCCTATGCACATCATCCTAAATTAATAAGCACATTAGCGCTTGCCCGCGCCACGCTATACAAACATTTACGGGAGTTAAAAC  CGCAAGGAGGGCCATCAAATGGCtgaGCGGCCGC | *cas3*-*cse1* fusion insert in pCas3-Cse1 |
