# Supplementary figures and images for: Type I-E CRISPR-Cas System as a Defense System in Saccharomyces cerevisiae
Source: mSphere. 2022 Apr 27;7(3):e00038-22. doi: 10.1128/msphere.00038-22 (PMC9241507; doi:10.1128/msphere.00038-22)

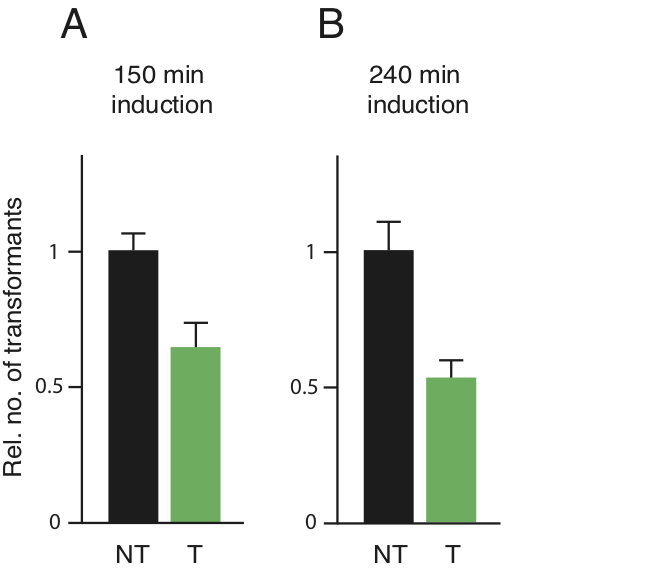

Supplement: FIG S1 [file msphere.00038-22-s0001.tif]

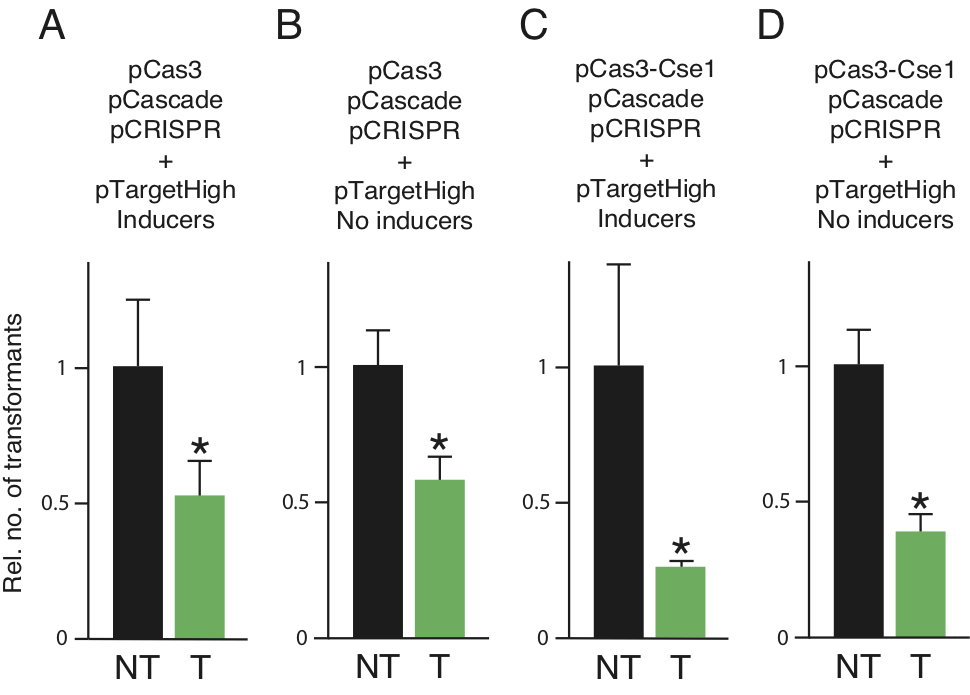

Supplement: FIG S2 [file msphere.00038-22-s0002.tif]

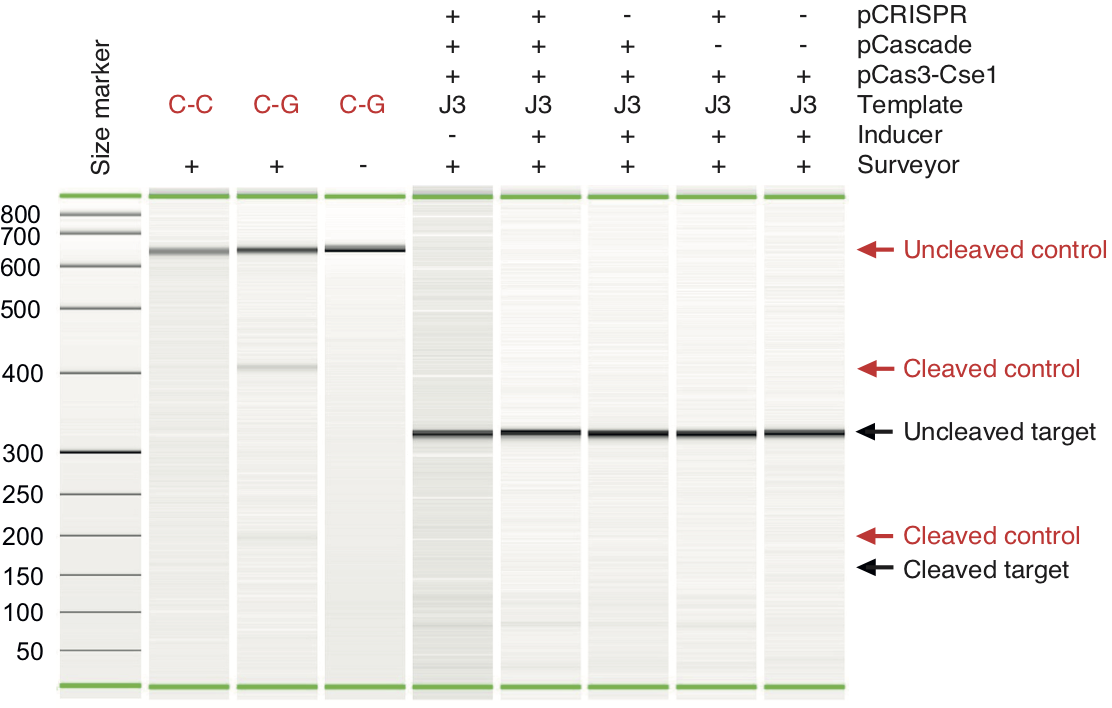

Supplement: FIG S3 [file msphere.00038-22-s0003.tif]

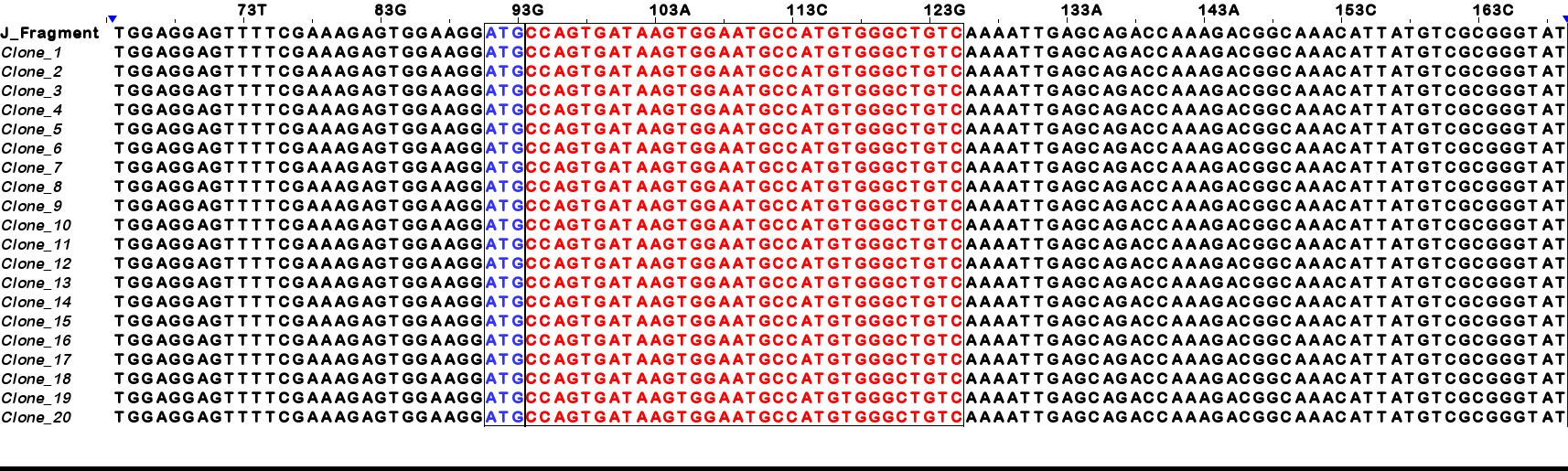

Supplement: FIG S4 [file msphere.00038-22-s0004.tif]
