# Supplementary material for: Type I-E CRISPR-Cas System as a Defense System in Saccharomyces cerevisiae
Source: mSphere. 2022 Apr 27;7(3):e00038-22. doi: 10.1128/msphere.00038-22 (PMC9241507; doi:10.1128/msphere.00038-22)
Supplement: TABLE S2 [file msphere.00038-22-s0006.docx]

Table S2: Transformation data for interference-active and control *S. cerevisiae* strains

| Strain’s plasmid content | Target cfu | Non-target cfu | Source data for figure |
| --- | --- | --- | --- |
| pCas3-Cse1  pCascade  pCRISPR | 5 | 13 | 3A |
|  | 3 | 27 |  |
|  | 4 | 17 |  |
| pCas3-Cse1  pCascade | 40 | 41 | 3B |
|  | 58 | 426 |  |
|  | 370 | 70 |  |
|  | 240 | 408 |  |
| pCascade  pCRISPR | 41 | 70 | 3C |
|  | 82 | 362 |  |
|  | 4 | 330 |  |
|  | 187 | 12 |  |
|  | 178 | 305 |  |
| pCas3-Cse1  pCRISPR | 1 | 13 | 3D |
|  | 16 | 12 |  |
|  | 6 | 25 |  |
| pCas3-Cse1 | 72 | 64 | 3E |
|  | 83 | 240 |  |
|  | 56 | 105 |  |
| pCascade | 78 | 83 | 3F |
|  | 48 | 181 |  |
|  | 54 | 92 |  |
