# Supplementary material for: Type I-E CRISPR-Cas System as a Defense System in Saccharomyces cerevisiae
Source: mSphere. 2022 Apr 27;7(3):e00038-22. doi: 10.1128/msphere.00038-22 (PMC9241507; doi:10.1128/msphere.00038-22)
Supplement: TABLE S3 [file msphere.00038-22-s0007.docx]

**Table S3**: List of strains

| Strain | Organism | Genotype | Source/reference |
| --- | --- | --- | --- |
| Top10 | *E. coli* | F– *mcrA*Δ(*mrr-hsdRMS-mcrBC*) Φ80*lacZ*Δ*M15* Δ*lacX74recA1araD139* Δ(*araleu*) *7697galUgalKrpsL* (Str^R^) *endA1nupG* | Invitrogen |
| MG1655 | *E. coli* | K-12 F^–^ λ^–^*ilvG*^–^*rfb*-*50rph-1* | Department collection |
| BL21AI | *E. coli* | F^-^ *ompT* *hsdS*_B_ (r_B_^-^ m_B_^-^) *gal dcm*  *araB*::T7RNAP-*tetA* | Invitrogen |
| W303 | *S. cerevisiae* | *MATa*/*MATαleu2-3*,-*112trp1-1can1-100ura3-1 ade2-1 his3-11,-15* [phi+] | R. J. Rothstein, Methods Enzymol 101:202-211, 1983, doi: 10.1016/0076-6879(83)01015-0 |
| BY418 | *S. cerevisiae* | *MATα ade2*∆*::hisG his3*∆*200leu2*∆*1 lys2*∆*202 met15*∆*0 trp1*∆*63 ura3-52* | C. B. Brachmann, *et al*., Yeast 14(2):115-132, 1998, doi: 10.1002/(SICI)1097-0061(19980130)14:2<115::AID-YEA204>3.0.CO;2-2 |
| BL21AI | *E. coli* | *F- ompT hsdSB (rB- mB-) gal dcm araB::T7RNAP-tetA* | Invitrogen |

Note: *S. cerevisiae* W303 were a kind a gift from Erik Johansson. *S. cerevisiae* BY418 was a kind gift from Tohru Yoshihisa.
