# Supplementary material for: Type I-E CRISPR-Cas System as a Defense System in Saccharomyces cerevisiae
Source: mSphere. 2022 Apr 27;7(3):e00038-22. doi: 10.1128/msphere.00038-22 (PMC9241507; doi:10.1128/msphere.00038-22)
Supplement: TABLE S4 [file msphere.00038-22-s0008.docx]

**Table S4**: List of primers

| Name | Sequence | Gene | Comment |
| --- | --- | --- | --- |
| TCYC-F-Ps | TCGATATCCTGCAGTTAGTTATGTCACGC | CYC1 | For cloning |
| TCYC-R-Ps | AAGCCTGCAGATTAAAGCCTTCGAGCG | CYC1 | For cloning |
| CASA-F-Xb | GCAGGAGAAATCTAGAAGCATACGAAGGAGAACAA | *cse1* | For cloning |
| CASA-R-Xb | CCAGTTGTTGCCAGTCTAGATATAAAGC | *cse1* | For cloning |
| CASB-F-Ec | ACATTTACGGGAATTCAAACCGCAAGGAG | *cse2* | For cloning |
| CASB-R-Ec | ATCAGAACATGAATTCTGATAAAGTTAGAC | *cse2* | For cloning |
| CASC-F-B | ACCACAAACAAGGATCCGTAAGGAAACCTT | *cas7* | For cloning |
| CASC-R | TCGCGCTCCAAGGACTGTATGGT | *cas7* | For cloning |
| CASD-F-Hd | CTGGGTTCGTAAGCTTGGCGAGGCGTGAA | *cas5e* | For cloning |
| CASD-R-Hd | TAATCCCTGGTGAAGCTTGTAAAGATCCCT | *cas5e* | For cloning |
| CASE-F-Sl | GAGAATGGTATGTCGACAAAGGAGGTATAGATGTATCTCA | *cas6e* | For cloning |
| CASE-R-Sl | GACGCGATCTTTGAGTCGACTGGGATTAAGGG | *cas6e* | For cloning |
| CasC3-F-Ec | AATAGCCCGCTGAATTCATCGATAATACT | *cas3* | For cloning |
| CasC3-R-Ec | CATTGATAACAAGAATTCCCGAAGTTATTTG | *cas3* | For cloning |
| DR003 | TCGGGCGAGCGATGATGCG | *lambda J* | For Surveyor assay and sequencing of survivors |
| DR004 | CATCGGCGTTTCATTCCCGTTT | *lambda J* | For Surveyor assay and sequencing of survivors |
| DR0015 | GGCCCGCTAGCGTCGGGCGAGCGATGATGCG | *lambda J* | For cloning |
| DR0016 | CGCGCGCTAGCCATCGGCGTTTCATTCCCGTTT | *lambda J* | For cloning |
| LAY001 | GAATTAGCTGATCATTAATAATAAGG | 4×J3 CRISPR | For cloning |
| LAY002 | GTCGCGGCCGCTTTGAAAATGG | 4×J3 CRISPR | For cloning |
| LAY003 | GAATTAGCTGATCaTTAATAATAAGG | CYC1 | For cloning, lower case letters indicate changes that introduce restriction sites required for cloning |
| LAY004 | GTCgcGgccGcTTTGAAAATGG | CYC1 | For cloning, lower case letters indicate changes that introduce restriction sites required for cloning |
| LA006 | GTTCAAATTTCGCAGCAGC | *Cas2* | For cloning |
| LA007 | TGTTACATTAAGGTTGGTGGGTTG | *477 CRISPR* | For screening for CRISPR expansion |
| LA008 | CAATTACAACCGACAGGGAG | *477 CRISPR* | For screening for CRISPR expansion |
| LA009 | GTGGGTTGTTTTTATGGG | *J3 CRISPR in* E. coli | For cloning |
| LA013 | TTtctaGAATTCGGTGGACG | *J3 CRISPR in* E. coli | For cloning |
| LA014 | GACAGCCCACATGGCATTCCACTTATCACTGG | *J3 spacer* | Probe for northern blot |
| LA017 | GGAGgAtCcGAAATGAGTATGTTGG | *Cas2* | For cloning |
| LA022 | AAGGAGgaATtCCATGACCTGGC | *Cas1* | For cloning |
| LA023 | CACGACCAgtcgACTCATTTCAGC | *Cas1* | For cloning |
| LA024 | tctcttcttcttaggGCTACTCCGATGGCCTG | *Cas1* | For adding NLS tag |
| LA025 | aaggtttgagtcgacAATGAGTATGTTGGTCGTGG | *Cas1* | For adding NLS tag |
| LA028 | aagaagagaaaggttAGTATGTTGGTCGTGGTC | *Cas2* | For adding NLS tag |
| LA029 | cttaggCATggatccTCAGCTACTCCGATGG | *Cas2* | For adding NLS tag |
| pLlacO-C | GTGCTCAGTATCTTGTTATCCG | *pZE12Luc vector* | For cloning |
| pZE-Xba | CGGAAAGTCCAAATTGTAATG | *pZE12Luc vector* | For cloning |
