# Supplementary material for: Type I-E CRISPR-Cas System as a Defense System in Saccharomyces cerevisiae
Source: mSphere. 2022 Apr 27;7(3):e00038-22. doi: 10.1128/msphere.00038-22 (PMC9241507; doi:10.1128/msphere.00038-22)
Supplement: TABLE S5 [file msphere.00038-22-s0009.docx]

**Table S5**: List of plasmids

| Name | Description | Sites | Primers | Source |
| --- | --- | --- | --- | --- |
| pBlueScript II SK (+) |  |  |  | Stratagene corp. |
| pRS423Gal | Galactose inducible expression vector, His3 selection |  |  | T. W. Christianson, *et al*., Gene 110(1): 119-122, 1992, doi: http://10.1016/0378-1119(92)90454-W |
| pRS424Gal | Galactose inducible expression vector, Trp1 selection |  |  | T. W. Christianson, *et al*., Gene 110(1): 119-122, 1992, doi: http://10.1016/0378-1119(92)90454-W |
| pRS425Gal | Galactose inducible expression vector, Leu2 selection |  |  | T. W. Christianson, *et al*., Gene 110(1): 119-122, 1992, doi: http://10.1016/0378-1119(92)90454-W |
| pRS426Gal | Galactose inducible expression vector, Ura3 selection |  |  | T. W. Christianson, *et al*., Gene 110(1): 119-122, 1992, doi: http://10.1016/0378-1119(92)90454-W |
| pYES2 | Yeast expression vector. Source of CYC1 terminator |  |  | ThermoFisher Scientific Inc. |
| pWUR397 | Source of Cas3 |  |  | S. J. J Brouns, *et al*. Science 321(5891):960-964, 2008, doi: 10.1126/science.1159689 |
| pWUR400 | Source of Cascade |  |  | S. J. J Brouns, *et al*. Science 321(5891):960-964, 2008, doi: 10.1126/science.1159689 |
| pWUR657 | Source of *cas3*-*cse1*fusion |  |  | E. R. Westra, *et al*., Mol. Cell. 46(5): 595-605, 2012, doi: 10.1016/j.molcel.2012.03.018 |
| pWUR630 | Source of 4×J3 spacer |  |  | E. Semenova, *et al*., Proc Natl Acad Sci U S A 108(25):10098-10103, 2011, doi: 10.1073/pnas.1104144108 |
| pWUR610 | Source of Lambda J gene fragment (17918–18250) |  |  | E. Semenova, *et al*., Proc Natl Acad Sci U S A 108(25):10098-10103, 2011, doi: 10.1073/pnas.1104144108 |
| pWUR477 | Source of non-targeting spacer |  |  | S. J. J Brouns, *et al*. Science 321(5891):960-964, 2008, doi: 10.1126/science.1159689 |
| pUDM101 | Gal1-10 from pRS425Gal sub-cloned in pBlueScript II SK (+) | EcoRI-BamHI |  | This study |
| pUDM102 | CYC1 from pYES2 sub-cloned in pBlueScript II SK (+) | PstI | TCYC-F-Ps  TCYC-R-Ps | This study |
| pUDM104 | *cse2* from MG1655 cloned in pUDM101 | EcoRI | CASB-F-Ec  CASB-R-Ec | This study |
| pUDM105 | *cse6e*from MG1655 cloned in pRS425Gal | SalI | CASE-F-Sl  CASE-R-Sl | This study |
| pUDM107 | *cse1*from MG1655 cloned in pUDM104 | XbaI | CASA-F-Xb  CASA-R-Xb | This study |
| pUDM108 | *cas7* from MG1655 cloned in pUDM105 | BamHI | CASC-F-B  CASC-R | This study |
| pUDM109 | *cse1-cse2* from pUDM107 cloned in pUDM108 | SacI |  | This study |
| pUDM110 | *cas5e* cloned in pRS425Gal | HindIII | CASD-F-Hd  CASD-R-Hd | This study |
| pUDM111  (pCascade) | CYC1-*cas5e*-CYC1 from pUDM315 subcloned in pUDM109 | NotI |  | This study |
| pUDM313 | CYC1 from pUDM102 sub-cloned in pUDM110 | SalI |  | This study |
| pUDM315 | CYC1 from pUDM102 sub-cloned in pUDM313 (to construct CYC1-*cas5e*-CYC1) | SpeI |  | This study |
| pUDMCas3  (pCas3) | *cas3* from MG1655 cloned in pRS423Gal | EcoRI | CasC3-F-Ec  CasC3-R-Ec | This study |
| pUDM113  (pCas3-Cse1) | *cas3*-*cse1*from pWUR657 sub-cloned in pRS423Gal | SpeI-NotI |  | This study |
| pRS424Gal_Cyc1 | CYC1from pUDM102 sub-cloned in pRS424Gal | SacII-SacI | LAY003  LAY004 | This study |
| pRS424Gal_Cyc1_4×J3  (pCRISPR) | 4×J3 spacer from pWUR630 cloned in pRS424Gal_Cyc1 | BamHI-NotI | LAY001  LAY002 | This study |
| pRS424Gal_J1-2_Cyc1 | J1-2 |  |  | This study |
| pRS424Gal-Cyc1_477 | Non-targeting spacer from pWUR477 cloned in pRS424Gal_Cyc1 |  |  | This study |
| pUDM416  (pCRISPR+Cas3-Cse1) | J1-2-CYC1 from pRS424Gal_J1-2_Cyc1 sub-cloned in pUDM113 | SalI |  | This study |
| pUDM421  (pCRISPR+Cas3) | J1-2-CYC1 from pRS424Gal_J1-2_Cyc1 sub-cloned in pUDMCas3 | BamHI-SacI |  | This study |
| pRS426_λ_350_  (pTargetHigh) | J fragment from pWUR610 sub-cloned in pRS426Gal | BamHI-HindIII |  | This study |
| pPS1739 | Low copy vector |  |  | P. Ferrigno, *et al*., EMBO J, 17(19):5606-5614, 1998, doi: 10.1093/emboj/17.19.5606 |
| pUDM117  (pTargetLow) | J fragment from pRS426_λ_350_ sub-cloned in pPS1739 | NheI | DR0015  DR0016 | This study |
| pLA002 | Minimal CRISPR array with -54 of the leader, two repeats and J3 spacer cloned in pZE12Luc for crRNA expression in *E. coli*. AmpR | Blunt, XbaI | LA009  LA013 | This study. |
| pLA005 | pRS426Gal_Cyc1 with Cas1 and Cas2 with SV40 NLS tags |  | LA006  LA017  LA022  LA023  LA024  LA025  LA028  LA029 | This study |
| pZE12Luc | Vector for *E. coli* CRISPR array. | Blunt, XbaI | pLlac-O  pZE-Xba | Lutz, R. and H. Bujard, Nucleic Acids Res, 1997.  **25**(6): p. 1203-10. |
| pWUR594 | Source of J3 spacer for *E. coli* CRISPR array. |  |  | Westra, E.R., *et al*., Mol Microbiol, 2010. **77**(6): p.  1380-93. |

Note: pRS423Gal, pRS424Gal, pRS425Gal, pRS426Gal were a kind a gift from Erik Johansson. pYES2 was a kind gift from Hans Ronne.
